# Periorbital edema as initial manifestation of chronic cutaneous lupus erythematosus

**Published:** 2012-07-02

**Authors:** Samar Erras, Laila Benjilali, Lamiaa Essaadouni

**Affiliations:** 1 Department Internal medicine, Mohamed VI Teaching Hospitals, Marrakech, Morocco

**Keywords:** Cutaneous Lupus, periorbital edema, Systemic Lupus Erythematosus, Anti-DNA antibodies, Morocco

## Abstract

Periorbital edema occurs frequently in dermatomyositis, but it has rarely been noted in systemic systemic lupus erythematosus. We describe a patient who developed bilateral periorbital edema and erythema as the sole manifestation of systemic lupus erythematosus.

## Introduction

Periorbital edema or eyelid edema may occur initially or in the course of a wide variety of diseases. The association with lupus erythematosus is rarely reported [1]. We present a case of lupus erythematosus in which the periorbital edema was the initial manifestation.

## Patient and case report

**Figure 1:**
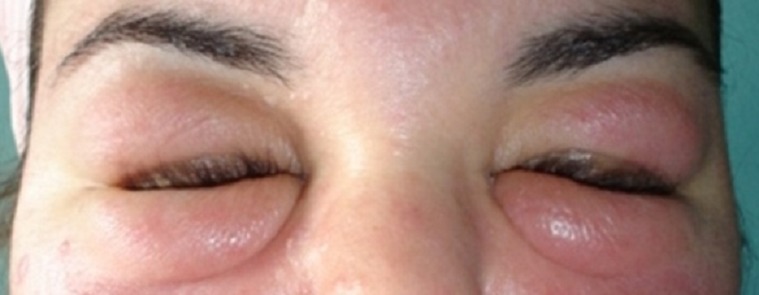
A 26-year-old woman with bilateral swelling and erythema of eyelids in chronic cutaneous lupus erythematosus

A 26-year-old woman was referred to the hospital because of a 3-years history of bilateral swelling and erythema of her eyelids (Figure 1). Initially the swelling occurred intermittently, but after 2 years it was permanent. Her past medical and family history was unremarkable; she reported no use of topical medications, cosmetics and contact lenses. Physical examination showed prominent swelling on a background of mild erythema involving the eyelids. There was no associated symptoms, including fever, pain, pruritus, or stinging. She was otherwise well and had no evidence of systemic involvement. Hematological, biochemical and serological tests for antinuclear antibody, anti-DNA, C1q, C2 and C1 inhibitor were normal. Chest X-ray and the sinuses X-rays were normal. The skin biopsy showed compact hyperkeratosis, diffuse but marked basal cell hydropic degeneration, and superficial and deep perivascular, as well as periadnexal lymphocytic infiltrate; It consistent with a diagnosis of chronic cutaneous lupus erythematosus (CCLE). The patient was treated with hydroxychloroquine 400 mg/day and prednisone was added at the initial dose of 60 mg/day. After one months of treatment, an edema was improved and there was discoloration of the erythema. And no recurrence has been observed in a follow-up period of 1 year.

## Discussion

Eyelids edema has been reported in patients with systemic lupus erythematosus [1]. Tuffanelli and Dubois reported a 0.1% incidence of periorbital edema as the presenting manifestation of systemic lupus erythematosus and an overall incidence of 4.8% [2]. It can be the presenting feature in patients with DLE [2], lupus panniculitis [3], lupus tumidus [4] and LE profundus [5], but other eye lesions can exist in CCLE and there are mostly asymptomatic and can lead to complications like loss of lid tissue, disorganization of mucocutaneous junction, loss of lashes, ectropion, entropion and symblepharon due to chronic inflammation of the deeper corium [6]. Chronic cutaneous lupus erythematosus can have more then 20 various clinical subtypes as discoid lupus erythematosus (DLE), lupus panniculitis, lupus tumidus, and chilblains LE, etc [7]. CCLE is more common in women, (female to male ratio ranges from 3,1 to 3,2) with the incidence peaking in the fourth decade of life [8]. The most common form of CCLE is classic DLE; it is characterized by inflammatory, scarring lesions mainly involving the head or neck, but also elsewhere, mostly on the photoexposed areas. The typical lesions in chronic discoid lupus erythematosus appear as round or oval erythematosus plaques with scales and follicular plugging [9].The sites most commonly involved are scalp, pinna of ears, eyebrows, eyelids, nose, chin, cheek, anterior thoracic region, upper portion of the arm, etc [8]. Cyran and et al. reported two similar cases and used the term chronic cutaneous lupus erythematosus to refer to the particular subset of LE into which their patients fall [2]. Seven of the 21 patients reported with cutaneous LE limited to the eyelids were treated with chloroquine (one of these with chloroquine in association with oral corticosteroids) and four with hydroxychloroquine [2]. Our patient responded to hydroxychloroquine and prednisone. Our case illustrates the difficulty in making the diagnosis of CCLE when the disease is limited to the eyelids; the average duration before correct diagnosis for eye lesions in CCLE, including our patient, is two-three years [10]. Histopathological findings represent an important clue, especially in our case, because the periorbital edema was the sole manifestation of CCLE [2].

## Conclusion

We suggest that every patient with persistent periorbital edema should undergo periodic clinical examination and histologic evaluation to identify recurrence and to institute early treatment in order to prevent complications.
